# Prevalence of cardiovascular health risk behaviors in a remote rural community of Sindhuli district, Nepal

**DOI:** 10.1186/1471-2261-14-92

**Published:** 2014-07-28

**Authors:** Raja Ram Dhungana, Surya Devkota, Mahesh Kumar Khanal, Yadav Gurung, Rajendra Kumar Giri, Ram Krishna Parajuli, Anup Adhikari, Suira Joshi, Barsha Hada, Arun Shayami

**Affiliations:** 1 Nepal Family Development Foundation, Kathmandu, Nepal; 2 Manmohan Cardiothoracic, Vascular and Transplant Centre, Institute of Medicine, Tribhuvan University, Kathmandu, Nepal; 3 Dhaka University, Dhaka, Bangladesh; 4 Auckland University of Technology, Auckland, New Zealand; 5 Ministry of Health and Population, Kathmandu, Nepal; 6 Youth Vision Central office Bhanimandal, Lalitpur, Nepal; 7 Tribhuvan University, Kathmandu, Nepal; 8 Nature Care Hospital, Baneshwor, Kathmandu, Nepal

**Keywords:** Behavioral risk factors, Cardiovascular diseases, Nepal, Rural community

## Abstract

**Background:**

Cardiovascular disease (CVD) is emerging as a public health menace among low and middle income countries. It has particularly affected the poorest. However, there is paucity of information about CVD risk factors profile among Nepalese rural communities where the majority of people live in poverty. Therefore, this study aimed to identify the prevalence of cardiovascular health risk behaviors in an outback community of Nepal.

**Methods:**

We conducted a descriptive cross-sectional study in Tinkanya Village Development Committee (VDC), Sindhuli between January and March, 2014. Total 406 participants of age 20 to 50 years were selected randomly. Data were collected using WHO-NCD STEPwise approach questionnaires and analyzed with SPSS V.16.0 and R i386 2.15.3 software.

**Result:**

The mean age of participants was 36.2 ± 9 years. Majority of participants (76.3%) were from lower socio-economic class, Adibasi/Janajati (63.1%), and without formal schooling (46.3%). Smoking was present in 28.6%, alcohol consumption in 47.8%, insufficient fruits and vegetables intake in 96.6%, insufficient physical activity in 48.8%; 25.6% had high waist circumference, 37.4% had overweight and obesity. Average daily salt intake per capita was 14.4 grams ±4.89 grams. Hypertension was detected in 12.3%. It had an inverse relationship with education and socio-economic status. In binary logistic regression analysis, age, smoking, body mass index (BMI) and daily salt intake were identified as significant predictors of hypertension.

**Conclusion:**

Present study showed high prevalence of smoking, alcohol consumption, insufficient fruit and vegetable intake, daily salt intake, overweight and obesity and hypertension among remote rural population suggesting higher risk for developing CVD in future. Nepalese rural communities, therefore, are in need of population-wide comprehensive intervention approaches for reducing CVD health risk behaviors.

## Background

Cardiovascular diseases (CVD) constitute a major public health problem in world accounting 30% of all global deaths [1]. The rapidly increasing CVD death toll is predicted to rise to 23 million by 2030 [2]. More importantly, CVD, once regarded as diseases of affluence, is now wildly spreading among low and middle income countries contributing more than three-quarters of all CVD deaths in the globe [3]. It is emerging as a major killer even in Nepal where mortality attributed to CVD has swiftly increased from 22% to 25% between 2004 and 2008 [4]. Besides, non-communicable diseases including CVD are exerting enormous burden on life of poor and marginalized people reducing labor productivity and increasing out of pocket expenditure; and ultimately creating more pressure on poor healthcare system and debilitating national economy [5,6].

Most cardiovascular diseases share common risk factors like tobacco use, physical inactivity, unhealthy diet, harmful use of alcohol, diabetes, high blood pressure and raised lipid. Among them, behavioral risk factors-unhealthy diet, physical inactivity, tobacco use and harmful use of alcohol, alone contributes 80% of coronary heart disease and cerebrovascular disease [4]. Smoking is estimated to cause nearly ten per cent of all CVD followed by physical inactivity (6%), and overweight and obesity (5%) [7]. Low fruits and vegetables intakes also caused death of approximately 16 million people [7]. Recent studies have reported high prevalence of behavioral related CVD risk factors in Nepal [8,9]. National NCD risk factors survey 2013 detected considerably high proportions of smoking (18.5%), alcohol consumption (17.4%), insufficient fruits and vegetables consumption (98.9%) and obesity (4%) among Nepalese [10].

Some studies conducted in rural Nepal, India, Malaysia and Nigeria indicated high prevalence of CVD risk factors [11-16]. They were also estimated to increase substantially in future [17]. These studies also point out a negative association of education and socio-economic status to smoking, alcohol consumption, hypertension [15] and lower fruits intake [13]. But, there is dearth of such studies particularly among Nepalese remote rural population.

As sufficient evidences are available to prove CVDs and other non communicable diseases have higher propensity to spread in low socioeconomic status leading life of the poorest to abysmal poverty [5,18], remote rural communities should also be tracked well on time for understanding the ongoing epidemiological transition. Therefore, the primary purpose of the study was to estimate the prevalence of cardiovascular health risk behaviors among people living in remotely located area of Eastern Nepal where poor hygiene and sanitation still remains a prevailing problem. We also assessed the association between CVD risk factors and hypertension. Study finding would be useful for identifying the extent of the problem and implementing CVD prevention programs among similar communities in Nepal.

## Methods

### Study site

This was a descriptive, community based, cross-sectional study conducted between January and April 2014 in Tinkanya Village Development Committee, Sindhuli, Nepal. Study site is an extremely hilly, remote, rural community where mostly indigenous people live. Developmental markers like roads, electricity and health facilities still remain almost nonexistence. A part from livestock and farming, remittance is the major source of livelihood. Most working age men have migrated to city/abroad for better employment.

### Study population and sample size

The community contains 805 households including 4169 population residing in nine Wards [19]. We divided nine Wards into three clusters and organized a screening camp on each clusters separately. Community leaders, teachers and elderly persons were mobilized for encouraging eligible people for their voluntarily participation.

We calculated the sample size using one sample situation of estimating population proportion with specified absolute precision formula [20]. The estimation of population prevalence of risk factors was considered as 50% with equal percent (5%) of allowable error, level of significance and non response rate. Based on predefined sample size and sampling method, we included 406 participants, almost equal numbers from each cluster, who provided informed consent to involve in study. Every third registered participants who met the inclusion criteria of having age between 20 and 50 years and without an established CVD were enrolled for the study.

### Ethical consideration

Study protocol was reviewed and approved by Institutional Review Board of Institute of Medicine, Kathmandu. We obtained the verbal consent from all participants after detailed explanation of research purpose, and assurance of maintaining privacy and confidentiality. Free heath checkup and medicines were provided to the participants based on their health problems. Those who needed further treatment were referred to higher centers. A successive awareness camp was also organized for educating people on CVD risk factors.

### Study tool

We followed the WHO-NCD STEPwise approach to surveillance questionnaires for collecting demographic information, behavioural and anthropometric measurements [21]. It contains core and extended questionnaires related to smoking, alcohol consumption, fruits and vegetables intake, and physical activities [22]. STEP 2 core provides the measure of height, weight, waist circumference and blood pressure. We also added some structured questionnaires relevant to CVD risk factors into this tool.

### Data collection

All data enumerators were trained in research protocol, interviewing and measuring the variables. They were supervised by field co-coordinators experienced in national NCD risk factors surveillance. Research tool was appropriately modified after undertaking a pretest.

Weight was measured using CAMRY weighing machine. Height and waist circumference were noted in centimeter using Johnson’s non-stretchable measuring tapes. Waist circumference was measured in between the lower rib and anterior superior iliac crest. Doctor’s Aneroid Sphygmomanometer (BP Set) was used for recording blood pressure. We recorded three readings of systolic and diastolic blood pressure in five minutes interval over right arm and the latter two were averaged for final score. Socio-economic status was assessed using revised Kuppuswamy’s scale [23]. Average salt intake per person per day was calculated in four steps. First, the total days that one family could sufficiently use one kilogram of salt (one packet) were counted. If same salt was used for cattle, that amount was also roughly estimated and reduced from it. Then, the total salt consumption was divided by total days it was used and number of family members who were sharing the same kitchen in order to derive salt intake per person per day. Finally, it was averaged to calculate average salt intake per capita per day.

Standard operational definitions were adopted for behavioral, anthropometric and clinical measurements to ensure uniformity and minimize error. Smoking and alcohol consumption were considered as present if participants had had habit of taking them until the past 30 days of interview. Current smokers were defined as those reported smoking any tobacco product within last 30 days. Person smoking daily was regarded as current daily smoker. Similarly, current alcohol drinkers were labeled for those who engaged in alcohol drinking within last 30 days. In the same way, current episodic heavy drinking was considered as five and more drinks on any day in past 30 days. One standard drink (13.6 gm of pure alcohol) was equivalent to consuming 341 ml of beer or Zaand or Tongba; and 43 ml of local Raksi [9].

Fruits and vegetables intake for at least five portions (400 gm) of a day was regarded as sufficient [24]. One serving of vegetable was considered to be one cup (250 ml) of raw green leafy vegetables and ½ cup (125 ml) of cooked or chopped raw vegetables. One whole medium sized fruits like apple, banana or orange and ½ cup of chopped, cooked, canned fruit or ½ cup of fruit juice was in general considered as one portion [24]. Amount was estimated by using show cards. Sufficient physical activity was defined as at least 150 minutes moderate physical exercise in a week. Body mass index (BMI) was calculated and categorized as normal (<25.0 kg/m^2^), overweight (25.0– < 30.0 kg/m^2^), and obese (≥30.0 kg/m^2^) [25,26]. Based on Asian criteria, abdominal obesity was defined as waist circumference ≥ 90 centimeters in men and ≥ 80 centimeters in women [27]. The diagnostic criterion for hypertension was set systolic blood pressure ≥ 140 mmHg and/or a diastolic blood pressure ≥ 90 mmHg as recommended by Joint National Committee-VII [28]. The persons who were using antihypertensive medicine were also listed under the category of hypertension.

### Data management and analysis

Data were compiled, edited and checked to maintain consistency. Repetitions and omissions of data were corrected before coding and entering them in Epidata V.2.1. Recorded data were, then, exported to SPSS V.16.0 for further analysis. R i386 2.15.3 was used for comparing proportions and calculating confidence interval.

Descriptive statistics was used to identify the distribution of risk factors among socio-demographic characteristics. Chi-square and independent t test were conducted for comparing proportions of categorical and mean of continuous variables. All tests were two-tailed and p < 0.05 was considered statistically significant. Bivarate and multivariate analyses were conducted by binary logistic regression. Categorical variables were coded appropriately before entering them into model. Hypertension was considered as a dependent variable with dichotomous outcomes: 0 = Absent, 1 = Present. Other variables were coded as:- a) smoking: 0 = No, 1 = Yes, b) alcohol consumption: 0 = No, 1 = Yes, c) fruits and vegetables intake: 0 = sufficient, 1 = insufficient, d) physical activity: 0 = sufficient, 1 = insufficient.

Separate bivariate logistic regression analyses were conducted for independent variables. Correlation matrix was calculated to investigate their interrelationship. Variables those were identified as significant predictors in bivariate analyses were entered in multivariate model. Independent variables having significant correlation with each other were further investigated for interaction in multivariate analysis. The final multivariable model included age, smoking, alcohol consumption, salt intake and BMI. Waist circumference was dropped out from the multivariable model because of collinearity with BMI.

## Result

### Demographic characteristics

This study contained total 406 participants, including 176 (43.3%) male and 230 (56.6%) female. The mean age was 36.2 ± 9 years, with statistically non-significant (t = 2.086, df = 404, p = 0.38) mean difference of 1.879 years between male and female. Majority of participants belonged to Adhiwasi/Janajati (indigenous group) (63.1%), Hindu (80.3%), and were married (86.7) (Table 1). Most respondents (46.3%) had not been to school, were involved in household activities (62.6%) and had lower socio-economic status (76.3%) (Table 1).

**Table 1:** Distribution of socio-demographic characteristics by gender

**Socio-demographic characteristics**		**Gender**		**Total no. (%)**	**P value**
		**Male no. (%)**	**Female no. (%)**		
Age group (in years)	20-30	38 (9.4)	82 (20.2)	120 (29.6)	.007
	31-40	76 (18.7)	76 (18.7)	152 (37.4)	
	41-50	62 (15.3)	72 (17.7)	134 (33)	
Ethnicity	Brahman and Chhetri	82 (20.2)	40 (9.9)	122 (30)	.000
	Adhiwasi/Janajati	80 (19.7)	176 (43.3)	256 (63.1)	
	Dalits	14 (3.4)	14 (3.4)	28 (6.9)	
Religion	Hindu	153 (37.7)	173 (42.6)	326 (80.3)	.003
	Buddhist	23 (5.7)	57 (14)	80 (19.7)	
Marital status	Married	156 (38.4)	196 (48.3)	352 (86.7)	.315
	Unmarried	20 (4.9)	34 (8.4)	54 (13.3)	
Education level	No formal education	50 (12.3)	138 (34)	188 (46.3)	0.000
	Lower than primary	24 (5.9)	34 (8.4)	58 (14.3)	
	Primary education	46 (11.3)	26 (6.4)	72 (17.7)	
	Secondary and high education	56 (13.8)	32 (7.9)	88 (21.7)	
Occupation	Government employee	14 (3.4)	6 (1.5)	20 (4.9)	0.000
	Non-governmental employee	8 (2)	4 (1)	12 (3)	
	Self employed	64 (15.8)	18 (4.4)	82 (20.2)	
	Unpaid volunteers	10 (2.5)	0	10 (2.5)	
	Students	2 (0.5)	18 (4.4)	20 (4.9)	
	Household activities	74 (18.2)	180 (44.3)	254 (62.6)	
	Retired	0	2	2(.5)	
	Unemployed	2 (0.5)	2 (0.5)	4 (1)	
	Unemployed (disabled)	2 (0.5)	0	2 (0.5)	
Socio-economic status (KUPPUSWAMY SCALE)	Lower	26 (6.4)	96 (23.6)	122 (30)	0.000
	Upper lower	90 (22.2)	98 (24.1)	188 (46.3)	
	Lower middle	34 (8.4)	30 (7.4)	64 (15.8)	
	Upper middle	26 (6.4)	6 (1.5)	32 (7.9)	
	Upper	0	0	0	

### Prevalence of cardiovascular risk factors

Among total participants, 28.6% people were smoking until last 30 days of interview and 27.1% of people were smoking daily. Smoking rate was significantly higher among 41–50 age-group, married, illiterate (no formal education) and lower socio-economic status than others (Table 2). Among smokers 64.6% people started smoking before 15 years of age and 63.8% did not even think to give it up. Nevertheless, almost all (91.6%, 406) participants unanimously replied that smoking is injurious to health. Nearly 2/3 respondents (70 .4%) believed smoking and second hand smoke caused respiratory and cardiac problems.

**Table 2:** Prevalence of cardiovascular risk factors

**Characteristics**	**Smoking**	**Alcohol consumption**	**Insufficient fruits and vegetables intake**	**Insufficient physical activity**	**Overweight and obesity**	**Hypertension**
Total	28.6 (24.2-33)	47.8 (42.9-52.7)	96.6 (94.8-98.4)	48.8 (43.9-53.7)	37.4 (32.7-42.1)	12.3 (9.1-15.5)
Age in years						
20-30	16.7	31.7	98.3	55	25	3.3
31-40	28.9	46.1	93.4	39.5	50	9.2
41-50	38.8	64.2	98.5	53.7	34.3	23.9
P value	0.0005	0.0001	0.028	0.014	0.0001	0.0001
Gender						
Male	26.1	52.3	96.6	53.4	39.8	14.8
Female	30.4	44.3	96.5	45.2	35.7	10.4
P value	0.34	0.11	.99	0.1	0.39	0.18
Caste						
Brahman/Chhetri	26.2	31.1	100	47.5	47.5	16.4
Adhibasi/Janajati	29.7	56.2	94.5	51.6	32	11.7
Dalits	28.6	42.9	100	28.6	42.9	0.0
P value	0.7850	0.0001	0.014	0.06	0.01	0.2
Religion						
Hindu	26.7	43.6	98.5	50.6	40.2	12.3
Buddhist	36.2	65	88.8	41.2	26.2	12.5
P value	0.089	0.005	0.001	0.133	0.02	0.95
Marital status						
Married	30.7	51.7	96.6	45.5	39.2	13.1
Unmarried	14.8	22.2	96.3	70.4	25.9	7.4
P value	0.016	0.0001	.99	0.0006	0.7	0.33
Education						
No formal education	44.7	58.5	97.9	45.7	38.3	14.9
Lower than primary education	17.2	55.2	93.1	37.9	27.6	13.8
Primary education	8.3	36.1	97.2	50	36.1	8.3
Secondary and Higher education	18.2	26	95.5	61.4	43.2	9.1
P value	0.0001	0.0001	0.32	0.0286	0.2890	0.3640
Socio-economic status						
Lower	42.6	57.4	96.7	54.1	63.9	14.8
Upper lower	27.7	42.6	98.9	42.6	68.1	12.8
Lower middle	18.8	53.1	96.9	50	53.1	9.4
Upper middle	0	31.2	81.2	62.5	43.8	6.2
P value	0.001	0.012	0.001	0.8	0.02	0.5

Total 194 (47.8%) participants had habit of drinking alcohol. Among them, 14.3% people were taking alcohol daily. Comparatively people without formal schooling, of age 41–50 years, from indigenous group and having low socio-economic status had significantly high indulgence to alcohol than others (Table 2). The average pure alcohol consumption was 110 gm with maximum limit to 400 gm. Among alcohol users, 62.2% (25.1% in total respondents) consumed equal or more than five standard drinks at any day during last month.

Sufficient fruits and vegetables consumption (≥5 servings/day) was lacking in 96.6% of respondents. Mean number of days of fruits and vegetables consumed per week were 2.4 and 4.3 days respectively. In the same way, mean number of fruits and vegetables servings per day were 0.48 and 1.05. Mostly illiterate (no formal education), socially adjudged higher caste group (Brahman and Chhetri) and upper lower class people had significantly high prevalence of inadequate amount of fruits and vegetables consumption (Table 2). More than two third (75.9%) of respondents were using vegetables oil (mustard, soybean, sunflower oil) for cooking purpose. But, small proportion of people (2%) occasionally utilized animal fat in replacement of vegetable cooking oil. Daily salt intake per person in an average was 14.4 gm ±4.89 gm. Large number of people (11.2%) were still beyond the coverage of iodized salt. They were using un-iodized raw salt. Though the study population was residing in hilly terrain, nearly half of respondents (48.8%) insufficiently (≤5 days per week) involved in moderate physical activity. People with age between 20 and 30 years, and having higher education were involving in significantly less physical activity. Though there was no significant difference in physical activity level by gender and socio-economic status, comparatively higher percentage of male and upper middle class people had low physical activity than others (Table 2). Mean body mass index and waist circumference were 24.5 kg/m^2^ and 79.8 cm respectively. Overweight and obesity (BMI ≥ 25 kg/m^2^) was present in 37.4% of people. It was relatively high in 31–40 age group and lower class population (Table 2). Over weight and obesity, separately, were observed in 29% and 8.4% of respondents. Abdominal obesity marker-waist circumference was above normal cut off point (≥80 cm in female, ≥90 cm in male) in 25.6% of participants.

### Hypertension

The estimated prevalence of hypertension was 12.3% (9.1-15.5%). It had inverse relationship with education and socio-economic status. Hypertension was relatively higher in low socio-economic level, people without any formal education, married, male and the oldest age group than others. In bivarate analysis, hypertension was significantly associated with age (β = .08, df = 1, p = .000), smoking (β = 1.164, df = 1, p = .000), alcohol consumption (β = .75, df = 1, p = .016), salt intake((β = .097, df = 1, p = .004), and BMI (β = .112, df = 1, p = .006). These variables were entered in binary logistic regression model for multivariate analysis. All four variables, except alcohol consumption were identified as significant predictors of hypertension (Table 3). Hosmer and Lemeshow Test for goodness of fit was not statistically significant (p = 0.106) with fairly large pseudo R square (Nagelkerke R Square = 0.222).

**Table 3:** Multivariate analysis for hypertension

**Variables**	**Category**	**B**	**P value**	**Exp(B)**	**95% C.I. for EXP(B)**	
					**Lower**	**Upper**
Constant		−10.255	.000	.000		
Age (years)		.093	.000	1.098	1.049	1.148
Smoking	No	Reference				
	Yes	.826	.017	2.285	1.161	4.498
Alcohol consumption	No	Reference				
	Yes	.405	.250	1.500	.752	2.993
Salt intake (grams)		.130	.001	1.139	1.056	1.230
BMI (kg/m^2^)		.085	.046	1.088	1.001	1.183

Multivariate analysis underscored smoking as a strong predictor of hypertension. Smoker was 2.285 times more likely to have hypertension than non-smoker (Table 3). Following smoking, salt intake remained to be second strong explanatory variables for hypertension. Every one gram more intake of salt increased the probability of being hypertensive by almost 14%. Similarly, age was also positively associated with hypertension (Table 3). If person was getting older, there was more likelihood of getting hypertension. Excessive fat deposition on body also had high tendency to increase blood pressure. One unit addition in body mass index could heighten the risk by 0.088 times for being hypertensive, holding other predictors constant.

## Discussion

This study presented the CVD risk factors burden among rural population in Eastern Nepal and found comparatively high prevalence of smoking, alcohol consumption, insufficient fruits and vegetables consumption, salt intake, overweight and obesity, and hypertension.

The prevalence of current smoking was 28.6% which was slightly lower than the result presented by Vaidya et al. (33.3%) [12] and WHO referred age standardized smoking rate (30%) in Nepal [29]. But it was comparatively higher than prevalence (18.5%) demonstrated by nationwide NCD risk factors survey 2013 [10]. Particularly, proportion of female smokers was larger than that of male. Reason could be presence of predominant numbers of 41–50 years aged female participants in study. It was found that respondents with age between 41 and 50 years were more indulgent to smoking than other age groups.

Current study showed that one out of two participants (47.8%) had a history of taking alcohol within last 30 days. But NCD risk factors survey 2013 in Nepal had only detected one in every six people (17.4%) as a current alcohol drinker [10]. Notably higher numbers of indigenous people at study site had a strong influence on prevalence of alcohol consumption. The predominance of lower socio-economic class and less educated people also stimulated a surge of alcohol consumption rate.

This study revealed high percentage (96.6%) of fruits and vegetables consumption less than the minimum recommended five daily servings per day among study population, which was also consistent with results of other national studies [9,10]. However, it was substantially higher than that of low- and middle-income countries (77.6% of men and 78.4% of women), India (74%) and Bangladesh (76.3%) [30]. Complete reliance on seasonal fruits and vegetables might be a big hurdle in sufficient fruits and vegetables intake in study site. Similar to low amount of fruits and vegetables consumption, excess daily salt intake per capita (14.4 gm) also persisted as a great health concern among study population. It was three times higher than WHO recommended amount of salt intake (5 gm) and nearly double than mean dietary salt intake (8.5 gm) by urban south Indian population [31]. But, data available from other studies like a research in Khotang, Nepal (10–13 gm per person per day) [32], Indian Council of Medical Research( ICMR) study in India (13.8 gm per person per day) [33] and National Heart Foundation Hospital and Research Institute (NHFH& R) study in Bangladesh (17 gm per person per day) [34] indicate that high salt consumption is rather a regional problem.

Current study findings suggested that higher education and upper socio-economic status were negatively associated with physical activity level. Most strikingly, this study also found that more males than females were insufficiently involving in moderate physical activity. It was because the community had only scarce number of young males. Most of them had either moved to city or abroad leaving only elderly and female residents in community. Furthermore, this unequal distribution of demographic characteristics (mainly age and gender) might be influential on high prevalence of overweight and obesity (37.4%) among study population. Though proportion of overweight (29%) and obese (8.4%) persons were similar to earlier rural community based study [12], it was relatively much higher than prevalence reported on both NCD risk factor surveys conducted in 2007 (obesity- 1.7%) [9] and 2013 (overweight-21.6%, obesity-4%) [10]. Prevalence of obesity in current study also strongly contrasts with WHO estimated age standardized obese person’s proportion in Nepal (1.5%) [29]. This rejects the assumption that rural populations are physically more active and less obese than urban dwellers. Result also substantiates the idea that rural population should also be provided equal attention on CVD prevention programs.

Compared to other risk factors, hypertension (P =12.3%) did not seem as a burgeoning burden among study population. Its prevalence was lesser than WHO (27.8%) [29], and NCD survey 2007 (21.5%) [9] and 2013 (25.7%) [10] estimates. In a recent community based study conducted among women in rural community of eastern Nepal, prevalence of hypertension was only 3.3% [35], which is largely differed from another hospital based study that identified 34% people had hypertension out of total admitted patients [36]. Such inconsistency in findings necessitates a nationwide population survey for estimating prevalence of hypertension.

The present study identified smoking, excess salt intake and increase BMI as strong predictors of hypertension. Preponderance of evidences has already been there to establish smoking, high salt consumption and obesity as risk factors for hypertension [7,37,38]. A study conducted in rural Nepal also identified that increased salt intake and high body mass index contributed for three fold rise in prevalence of hypertension during 25 years [17]. In spite of established relationship between alcohol and hypertension [39,40], our study could not determine alcohol consumption as a significant explanatory variable for hypertension. We have no clear explanation for it. However, this study corroborated the earlier study findings [13,15] that prevalence of smoking, alcohol consumption, fruits and vegetables intake and hypertension were comparatively high among less educated and low socio-economic status group.

## Conclusion

From the current research findings, it can be inferred that remote rural community has also been infiltrated by high prevalence of behavioral related CVD risk factors. Cases of CVD are likely to increase in near future if these risk factors continue unabated. Therefore, community based CVD prevention program is the need of the day.

### Study limitation

Significantly high numbers of female and indigenous group participants were involved in study. However, it was unintentional and was true representation of the present community where mainly women and indigenous people were living. Study findings should cautiously be generalized to other remote rural communities. Method used for measuring daily salt intake per person could be inaccurate. A more accurate way to determine dietary sodium, such as 24-hour urine collection, is warranted to confirm our findings. Similarly, scale used for determining socio-economic status has not yet been validated in Nepal.

## Competing interest

The authors declare that they have no competing interests.

## Authors’ contributions

RRD designed the study, analyzed and interpreted the data and prepared the first draft of the manuscript. AS, SD, MKK, RKP, YG provided concept and contributed in study design. AA, SJ, BH contributed to acquisition and compilation of the data. RKG performed revision of the manuscript. All authors read and approved the final manuscript.

## Pre-publication history

The pre-publication history for this paper can be accessed here:

http://www.biomedcentral.com/1471-2261/14/92/prepub
